# Farming fish in the sea will not nourish the world

**DOI:** 10.1038/s41467-020-19679-9

**Published:** 2020-11-16

**Authors:** Ben Belton, David C. Little, Wenbo Zhang, Peter Edwards, Michael Skladany, Shakuntala H. Thilsted

**Affiliations:** 1 Department of Agricultural, Food and Resource Economics, Michigan State University, East Lansing, MI USA; 2 WorldFish, Bayan Lepas, Pulau Pinang Malaysia; 3 Institute of Aquaculture, University of Stirling, Stirling, Scotland UK; 4 College of Fisheries and Life Science, Shanghai Ocean University, Shanghai, 201306 China; 5 School of Environment, Resources and Development, Asian Institute of Technology, Khlong Luang, Pathum Thani Thailand; 6 Department of Criminology, Anthropology, and Sociology, Cleveland State University, Cleveland, OH USA

**Keywords:** Conservation biology, Marine biology, Interdisciplinary studies

## Abstract

Recent literature on marine fish farming brands it as potentially compatible with sustainable resource use, conservation, and human nutrition goals, and aligns with the emerging policy discourse of ‘blue growth’. We advance a two-pronged critique. First, contemporary narratives tend to overstate marine finfish aquaculture’s potential to deliver food security and environmental sustainability. Second, they often align with efforts to enclose maritime space that could facilitate its allocation to extractive industries and conservation interests and exclude fishers. Policies and investments that seek to increase the availability and accessibility of affordable and sustainable farmed aquatic foods should focus on freshwater aquaculture.

## Abstract (web summary)

Marine aquaculture is widely proposed as compatible with ocean sustainability, biodiversity conservation, and human nutrition goals. In this Perspective, Belton and colleagues dispute the empirical validity of such claims and contend that the potential of marine aquaculture has been much exaggerated.

## Introduction

A rapidly growing and high-profile science and policy literature presents marine aquaculture as the forefront of environmentally responsible food production^1–7^. Futurologists and aquaculture advocates have long promoted ‘farming the seas’ as a technological and spatial fix for apparent constraints to terrestrial food production. We identify a ‘new wave’ of marine aquaculture literature that diverges from this narrative in three important ways. (1) It replaces ad hoc claims about the potential of marine aquaculture with a coordinated set of narratives. In combination, these rebrand marine finfish farming from an ecologically damaging and inequitable activity, to one potentially compatible with sustainable resource use, conservation, and human nutrition goals. (2) It locates the future of marine aquaculture in offshore waters; a vast zone previously beyond the bounds of existing technology, now positioned as a new frontier for expansion^8–10^. (3) It is advanced by new coalitions of actors, including conservation NGOs and fisheries scientists which previously tended to oppose marine aquaculture.

We make the case that this shift in the contemporary scientific debate stems from an alignment with the emerging policy discourse of ‘blue growth’^11,12^ and provide a two-pronged critique.

First, we contend that the ‘new wave’ of literature is empirically inaccurate regarding the potential of marine aquaculture to contribute to global food and nutrition security and environmental sustainability, especially with respect to offshore finfish farming. The economics of operating in exposed offshore environments necessitate production of high market value, carnivorous fish species that would remain inaccessible to low-income consumers and the food-insecure, even given significant technological advances.

Second, we suggest that contemporary science and policy narratives around marine aquaculture, and the alliances of actors that propagate them, contribute—through their alignment with the blue growth policy agenda—to a global drive to create and enclose maritime territory through the extension of private property rights that would accelerate allocation of the oceans to extractive industries and conservation interests. Marine enclosures facilitated by ‘blue growth’ could exclude and displace coastal fishers, who presently account for the vast majority of ocean users^13,14^, potentially eroding the significant contributions that small-scale fisheries make to human nutrition^14,15^.

Our critique is based on inductive analysis of key themes in recent peer-reviewed literature and policy documents on marine aquaculture, identified through an extensive review. Throughout this paper, we present statements and quotes from the literature to illustrate how narratives around mariculture are constructed using claims that draw on, and advance, the blue growth agenda.

The paper is structured as follows. First, we identify the major themes in recent marine aquaculture literature, and examine how these reproduce, and diverge from, the earlier literature. Second, we assess the empirical validity of recent claims about the potential of marine aquaculture to contribute to food and nutrition security and environmental sustainability. Third, we show how marine aquaculture is being reframed as compatible with conservation objectives. Fourth, we critically evaluate recent estimates of spatial potential for the expansion of marine aquaculture. Fifth, we explore how new alliances of actors formed around marine aquaculture contribute to a larger drive to commodify the oceans.

We conclude that marine finfish aquaculture would largely fail to deliver the food and nutrition security and environmental sustainability gains claimed due to biological, technical, and economic constraints. On the contrary, the expansion of marine aquaculture may intensify pressure on marine resources, and fuel exclusionary and inequitable social outcomes similar to those associated with ‘green grabs’ by conservation organizations on land^16,17^. We contend that the future of most farmed aquatic food production—including that with the greatest potential to contribute to food and nutrition security and equity goals—lies not in the sea, but on land. At sea, small-scale capture fisheries have greater potential to meet food and nutrition security and equity goals than most forms of marine finfish farming.

## (Re)imagining marine aquaculture

The idea that ‘farming the seas’ is an inevitable next step for humanity rapidly approaching limits to the growth of terrestrial food production is an old one. Quotes articulating a perceived need for marine aquaculture spanning almost six decades from 1952 to 2009, are presented in Table 1. All invoke urgent limits to terrestrial food production, counterposed with the yet-to-be-realized spatial fix of expansion into marine environments.

**Table 1:** Historical claims about the future of food production.

Statement	Date
• The oceans may someday produce a greater proportion of the food consumed by humans than is grown on the land^96^.	1952
• We are now entering…the era of mariculture… As the world’s population expands, land farming will be increasingly unable to meet the ever greater demands for food^97^.	1970
• Following the cultivation of land for food, society must take the next step: largescale domestication of the ocean^98^.	2005
• [Mariculture] will constitute the next food revolution in human history^99^.	2009

This deterministic mix of Malthusian anxiety and biotechnological utopianism^18^ is also central to the new wave of marine aquaculture literature. Recent literature emphasizes—in broad terms—a global crisis, entailing a growing, seafood-hungry population, collapsing capture fisheries, freshwater scarcity, competition for agricultural land, and conflicts over coastal space. This scenario, it is claimed, will drive the future of aquatic food production into increasingly distant offshore regions of the oceans^7–9,19,20^. The effect of this crisis narrative is to frame a series of contextually specific and highly variegated processes as universal trends. To cite but one example, whereas global capture fisheries decline is often taken as a given, many ‘collapsed’ stocks have recovered^21^, and many tropical multi-species, multi-gear, artisanal fisheries—which particularly make important contributions to food and nutrition security—display a high degree of resilience to fishing pressure^22^.

We identify three ways in which the new literature diverges from the old. (1) It marshals additional layers of mutually reinforcing claims. (2) It involves a diverse mix of actors, including groups once broadly opposed to marine aquaculture. (3) It contributes to advancement of a new market-based political project^23^: ‘blue growth’.

‘New’ claims in the contemporary literature can be summarized as follows. First, (reversing earlier popular representations) rather than driving inequitable outcomes for human food and nutrition security, marine aquaculture “has the potential to benefit malnourished populations”^24^. Second, (also reversing earlier representations by environmentalists) marine aquaculture “could be one of the most ecologically sustainable forms of food production”^3^. Third, marine fish farms can be “compatible with conservation objectives”, including the establishment of marine protected areas (MPAs)^25^. Fourth, geospatial modelling reveals a “vast amount of space suitable for marine aquaculture”^5^. Fifth, an apparent disjuncture between the urgent need for marine aquaculture and its unrealized potential can be explained in terms of policy failures^8,26^ that can (and, normatively, should) be remedied through marine spatial planning^6^ and expansion of private property regimes at sea^3^.

Following sections of the paper elaborate on and critically evaluate each set of claims. We contend that the cumulative logic of these claims aligns with the blue growth agenda of deepening marine private property regimes, and that this goal underpins support for marine aquaculture by some conservation advocates and fisheries scientists.

## Food and nutrition security and environmental sustainability

In this section, we critically evaluate claims in the recent scientific and policy literature on the potential of marine aquaculture to contribute to food and nutrition security and environmental sustainability. We find that these claims fail to adequately account for the bio-economic characteristics of the organisms farmed and technologies required to produce them. As a result, they obscure the likely distributional consequences and environmental externalities of expanding production and exaggerate potential for sectoral growth.

Recent literature emphasizes food production as marine aquaculture’s core function. This is evident from papers and policy documents with titles such as, Global opportunities for mariculture development to promote human nutrition^24^, How can mariculture better help feed humanity?^27^, Food from the Oceans^7^, and The future of food from the sea^2,3^. Collectively, the literature asserts that increased production of farmed marine food has the potential to enhance seafood consumption by humans^3^. Although some publications have little to say about how this additional seafood would be distributed, others make explicit, though unelaborated, claims about its potential contributions to food and nutrition security (see Table 2).

**Table 2:** Recent statements about marine aquaculture, food and nutrition security, and environmental sustainability.

Food and nutrition security
• Increased mariculture production could help ameliorate global malnutrition^24^.
• Mariculture offers a crucial supply of protein, and… can support nutritionally vulnerable communities^1^.
• [Offshore finfish aquaculture] operations could be sited in developing countries to increase food security through income generation or increased access to seafood^9^.

Environmental sustainability
• Part of the growing interest in offshore aquaculture is the potential for improved sustainability. By moving farther offshore into the less protected ocean environment, open-ocean farming has the potential to reduce some of the many negative impacts associated with more nearshore practices and even create positive impacts through greater resource efficiency use^28^.
• Offshore aquaculture is increasingly viewed as a mechanism to meet growing protein demand for seafood, while minimizing adverse consequences on the environment^19^.
• Offshore mariculture aquaculture could enable increased seafood production and economic development while alleviating pressure on coastal ecosystems and wild fisheries^10^.

Representations of marine aquaculture as an environmentally sustainable form of food production draw on three sets of claims. (1) Seaweeds and most molluscs are extractive feeders, requiring little or no external feed inputs or supplementary nutrients^3,7^. (2) Siting marine fish cages in offshore environments exposed to strong currents and wave action minimizes point source pollution that can occur when farms are sited in protected nearshore environments^10,19,28^. (3) Improvements in feed formulation reduce the adverse environmental impacts of marine finfish production^3,7,29^ (see Table 2). We address these points in turn below.

First, the potential of seaweeds and filter-feeding molluscs (bivalves) to contribute significantly to food and nutrition security falls short of their attractive resource use profiles. Leading seaweed industry experts estimate that global production of seaweeds stands at about half the quantity reported by the Food and Agriculture Organization of the United Nations (FAO)^30^. Seaweed is currently eaten directly in a limited range of forms; principally as soups, soup stock, sushi wrap, salads, and dried snacks^31^. Direct seaweed consumption per capita in Japan (which has the largest consumption per capita globally, along with Korea) has been reported at around 5.3 g/capita/day (<2 kg/capita/year). This level of consumption has been stable for decades^32^. Seaweeds consumed directly as food are likely to remain a minor component of future diets, with only niche markets outside East Asia^33^ and some Pacific islands. Growth of global seaweed production over the past decade has been driven mainly by expanding Indonesian output, which is used mostly for industrial carrageenan extraction^34^. Seaweed extracts are used by the food industry as thickening agents and gels in ultra-processed foods, such as confectionary, ice cream, desserts, and processed meats^31^. These types of food are often of limited nutritional value and can contribute negatively to human health when consumed in excess^35^.

The contribution of bivalves to world food supplies is also smaller than FAO statistics (based on ‘wet weight equivalents’ that include the weight of inedible shell) would suggest (Table 3). Yield of edible meat from bivalves averages 17% of live weight, whereas, the edible yield of finfish averages 87%. The apparent contribution of bivalves to world food supplies is thus biased dramatically upward in direct comparisons with finfish^36^. There is potential to make greater use of mollusc extracts in highly processed functional foods^37^ but these are of limited relevance to global food and nutrition security. Bivalves are nutritious, and some farmed species such as mussels are relatively affordable. Nevertheless, demand in most markets, and thus contributions to food supply at the global scale, are presently rather limited. For these reasons, we confine our analysis in the remainder of this article to the production of marine finfish.

**Table 3:** Quantity and shares of global farmed production of aquatic organisms in freshwater, brackish water and marine environments^54^.

	Freshwater	Brackish water	Marine	Total
Quantity produced (million tonnes/year)^a^				
Aquatic plants	0.1	1.2	30.5	31.8
Crustaceans	3.1	5.0	0.3	8.4
Molluscs	0.2	0.1	17.1	17.4
Finfish	44.4	3.9	5.2	53.4
Total	47.9	10.1	53	111.1
Share of total, excluding aquatic plants (%)				
Crustaceans	4.0	6.3	0.4	10.7
Molluscs	0.3	0.1	21.5	22
Finfish	56.1	4.9	6.5	67.4
Total	60.3	11.3	28.4	100
Share of total, excluding aquatic plants, adjusting for edible weight^b^ (%)				
Crustaceans	2.2	3.4	0.2	5.8
Molluscs	0.1	0.0	5.5	5.6
Finfish	73.7	6.4	8.5	88.6
Total	75.9	9.9	14.2	100

^a^FAO reporting year 2017.

^b^Estimated using the following conversion factors: Crustaceans 0.36; Molluscs 0.17; Finfish 0.87^36^.

Second, exposed marine environments are far more technically challenging arenas for finfish farming than nearshore waters or land-based farms. Operational challenges and expenses increase with distance from shore, in the form of fuel costs and adaptations required to operate in high energy environments. Adaptations include reinforced and submersible cage structures, high levels of automation^38^, and large farm sizes to capture economies of scale^39,40^. As a result, R&D, fixed capital, and operating costs for open-ocean aquaculture are high^40^. This makes offshore aquaculture a relatively high-cost way of growing fish that is presently able to compete with inshore aquaculture only under limited circumstances^41^. We contend that the economics of offshore marine finfish aquaculture therefore necessitate industrial-scale cultivation of high market value species or products to offset production costs^39,40,42^.

Most high market value finfish species with potential for use in marine aquaculture are carnivorous. Their production requires feeds containing marine ingredients (fish meal and/or fish oil), or substitutes such as microalgae derivatives that are under development and are currently expensive. As a result, the cost of production of most marine fish is high relative to herbivorous/omnivorous freshwater fish, such as carps, catfish, and tilapia that readily assimilate high levels of cheaper terrestrial plant-based ingredients in diets^43^. The ability to breed and farm freshwater fish at low cost using relatively basic technologies^44^, makes them accessible to low- and middle-income consumers in countries with high levels of supply^45^, as well as to small- and medium-scale producers who benefit from farming them. The opposite is true of marine aquaculture, especially offshore, where high fixed and operating costs prevent participation by all but large investors. Job creation associated with offshore farms would be limited mainly to onshore fish processing which will increasingly be automated^46^.

We believe that, even allowing for improvements in technical efficiency and scale economies, most species promoted as candidates for marine finfish farming will be unable to compete on price with those that make up the bulk of freshwater aquaculture, and will not become accessible to low-income consumers. Salmon—the main finfish presently farmed in marine environments—provides a relevant example. Salmon farming has been subject to half a century of intensive R&D, great leaps in production efficiency, massive levels of industrial consolidation, and consistently declining real farmgate prices^47^, yet, high production costs mean that salmon remains a relative luxury, inaccessible to anyone outside the global middle class.

Planned investments in offshore aquaculture farms in China provide similar insight into the relationship between species choice, production economics, and distributional outcomes. Investments in offshore finfish farming projects totaling >USD 1 billion are planned, though none has been established to date^48^. Among ten major offshore aquaculture projects currently proposed in Chinese waters, three are slated to produce salmon, three to produce large yellow croaker, and the others a mix of luxury species that include Japanese seabass, puffer fish, tuna, and yellowtail amberjack^49^. Projects such as these, if eventually realized, would do little to contribute to global food and nutrition security, ameliorate malnutrition, or support nutritionally vulnerable people.

The high market value marine finfish species that would dominate offshore marine finfish aquaculture require diets containing high levels of omega-3 fatty acids to ensure fish health and welfare, and to maintain nutritional value for humans^50^. Until now, marine ingredients derived from wild caught fish have been the principal source of omega-3 fatty acids in fish feeds. Alternative algal based aqua-feed ingredients are currently under development^51^ and some are now reaching the market. It seems probable that these products will start to substitute for fish oils as a key source of omega-3 in feeds, but the investment required means that prices are likely to remain high, limiting their use to the diets of high value species^52^, and meaning that they are unlikely to serve as an alternative source of protein (i.e., as a fish meal replacement).

Proponents of marine aquaculture point to the role of improvements in feed formulation technology to overcome the ‘fish meal trap’, by the substitution of plant-source proteins such as soybean cake for fish meal in the diets of carnivorous fish^3,7,29^. However, improvements in the efficiency of marine ingredient use for farmed salmon^53^ have been achieved in combination with expensive selective breeding programs. Such genetic improvement programs are generally only economically viable for species produced in very large volumes. Salmon accounted for 30% of marine finfish aquaculture in 2018, among more than 100 marine finfish species farmed^54^. It cannot be assumed that other marine species will follow a similar development pathway to salmon or achieve equally large improvements in performance.

Significant growth of marine finfish aquaculture would therefore entail, at the minimum, a short-term increase in demand for marine feed ingredients. Current interest in intensifying the exploitation of mesopelagic fish stocks^7,55^ for use in aquaculture feeds underlines this point. Demand for marine feed ingredients has already altered marine ecosystems^56^. Increasing demand further has the potential to cause unsustainable levels of exploitation of some fish stocks^57^ and to compromise the food and nutrition security of nutritionally vulnerable populations if utilization of fish in aqua-feeds competes with human consumption^58^.

Although improvements in feed formulation and breeding have the potential to lessen dependence on conventional marine ingredients, inclusion of higher levels of terrestrial ingredients in feeds would also lead to burden-shifting and ecological trade-offs^59,60^. For instance, complete substitution of fish meal in shrimp diets with terrestrial feed ingredients has the potential to increase demand for freshwater by up to 63%, land by up to 81%, and phosphorous by up to 83%, meaning that the sustainability of substituting fish meal with plant ingredients should not be taken for granted^60^.

Third, life cycle assessment studies show consistently that the most significant adverse environmental impacts of fed aquaculture in both marine and freshwater environments derive from the global effects of feed production^61,62^. Recent mariculture literature tends to view land use and freshwater consumption primarily as a question of direct on-farm utilization. Expansion of fed aquaculture anywhere, including in offshore marine environments, would create tele-coupled demand for space, freshwater, and ecosystem services on land. Siting fish cages offshore may attenuate some of the worst effects of point source pollution emanating from farms^10,19^ but cannot address these global impacts, and may exacerbate some of them. For example, life cycle assessment indicates that fuel consumed by boats providing transport to and from offshore finfish farms can contribute a large share of overall environmental impact, concentrated particularly in the impact categories of cumulative energy demand, acidification, ozone layer depletion, photochemical oxidation, and global warming^63^.

## Conservation aquaculture

A dramatic shift has recently taken place in the way that conservation interests engage with marine aquaculture. Early literature on open-ocean aquaculture by environmental NGOs was highly critical, concluding that offshore finfish farms would replicate many of the environmental problems documented as affecting inshore salmon aquaculture at that time, such as effluent discharges, disease transmission to wild stocks, and fish escapes^64^. Escapes may be associated with interbreeding and competition between farmed and wild fish, and the introduction of non-native species^65^.

Recent literature has drawn attention to potential synergies between marine aquaculture and conservation^1,25,66–68^ and advocated enthusiastically for collaboration between these interest groups^66,68^.

Papers espousing this view rewrite longstanding narratives regarding marine aquaculture’s incompatibility with conservation and make the case for, “shifting the narrative and paradigm of aquaculture’s role in resource management”^66^. This movement was preceded by work contending that fish farms act as fish aggregating devices, meaning that creating no-fishing zones at farms would provide greater resilience for fish stocks where coastal aquaculture is practiced^68^. Recent work from conservation organizations suggests that aquaculture may be preferable to other uses proposed in multiple-use MPAs, including fisheries^25^.

The potential of marine aquaculture to facilitate exclusion of fishers from clearly defined areas of oceanic space, thereby facilitating protection for wild fish stocks, is attractive to some conservationists. This logic leads one set of authors to conclude that, “Over the last 20 years, marine aquaculture and conservation have been largely opposing forces. To secure the best deal for coastal fish stocks, fish-farmers and conservationists should work together”^68^. Other commentators are even more optimistic, stating, “The sheer potential of conservation aquaculture suggests a tale of redemption for aquaculture and opportunity for conservationists to bring in a new age of collaborative practices to address global issues”^66^. As we show in the final section of this paper, the blue growth policy agenda has provided impetus for these alliances to flourish.

## Potential for spatial expansion

A proliferation of recent studies has sought to demonstrate untapped potential of marine aquaculture through geospatial analyses and modelling exercises, incorporating parameters that include the physiological requirements and growth rates of candidate fish species, marine hydrography, habitat suitability, operational suitability, and competing uses of ocean space^5,6,10,20,69–71^. These studies claim to reveal the existence of vast areas of ocean well-suited to marine aquaculture, holding “remarkable potential”^10^ to produce quantities of seafood far in excess of current levels of demand, if utilized for this purpose (Table 4). Projections reported in these studies are products of the models deployed. One prominent global study derived its estimates by assuming no further economic, environmental, or social constraints to production^5^. This approach has been challenged with respect to finfish aquaculture on the basis that feed availability and feed costs would prevent further expansions of mariculture long before any ocean space limitations are reached^42^.

**Table 4:** Claims about potential for spatial expansion of marine aquaculture.

• [W]e find vast areas in nearly every coastal country that are suitable for aquaculture. The development potential far exceeds the space required to meet foreseeable seafood demand^5^.
• [I]f all areas designated as suitable in this analysis were developed… we estimate that ~15 billion tonnes of finfish could be grown every year—over 100 times the current global seafood consumption^5^.
• If only the most productive areas of the ocean were developed for fish aquaculture, the amount of seafood that is currently captured by all wild fisheries could be grown using less than 0.015% of the ocean’s surface area—a surface area less than Lake Michigan^5^.
• [T]he Caribbean’s potential to produce cobia from mariculture is extremely large, with an approximate total annual production from suitable sites of 43.1 MMT^10^.
• The Caribbean could match its current seafood production by farming cobia in just 179 km^2^ (0.006%) of its marine space^10^.

A study of the potential of cobia farming in the Caribbean found that projected production would drop from a headline result of 43.1 million tonnes/year to below 1.5 million tonnes/year if the price of cobia fell just 13% below its present market rate of USD 8.62 kg^10^. Even this lower bound estimate of potential production appears highly improbable in light of current figures. Producing 1.5 million tonnes/year of farmed cobia would equate to a supply increase several orders of magnitude over the current total global output of farmed and wild cobia^54^. We believe that such a large increase in production, even if realized over a long period of time, would likely drive down cobia prices far below the current market value, undermining the economic viability of the industry and limiting its scope for expansion long before projected production volumes were reached.

The magnitude of the discrepancy between the potential for marine aquaculture expansion that these studies claim to reveal, and the material realities that would confront attempts to increase production on such as scale, cast doubt on the empirical validity of these projections. Rather, we argue that studies such as these may serve to produce the following effects:

First, in combination with narratives that frame an imminent crisis in global fish supplies and position marine aquaculture as compatible with food and nutrition security, environmental sustainability and conservation objectives, they create a discursive terrain in which marine aquaculture appears possible, necessary, desirable, and ultimately, inevitable^72^.

Second, through the technology of geospatial mapping, they render the oceans ‘legible’^73^ and amenable to technical planning initiatives^74^ that facilitate the division and allocation of ocean space among ‘resource users’ and the provision of exclusive access rights, based on criteria that appear objective and a-political^75^.

## Policy failures

Marine offshore aquaculture proponents seek to explain the gap between apparently massive potential and lackluster development to date in terms of past policy failures^8,26^. A recent high-profile policy report poses the question “If the potential [for mariculture] is so large, why is our production so low?” and answers, “This large gap is likely driven in part by prohibitive regulatory barriers in many countries”^3^. ‘Policy failures’ are characterized in the literature as taking the form of: (1) excessive regulation based on the precautionary principle^2,3^; (2) inadequate or absent regulation, allowing unrestricted expansion of aquaculture with negative environmental externalities^2,3^; (3) complex, ambiguous and overlapping regulations that hamper investments^8^.

Absence of private property rights for the sea has been invoked as a key underlying institutional constraint, preventing farms from excluding other users from their production sites^76^, and discouraging investments, including R&D, that are necessary for the industry to grow^3^. Marine spatial planning utilizing geospatial information is framed as enabling the zoning of oceanic space for efficient allocation among resource users that—in combination with private property rights and streamlined permitting processes—would create ‘win-win’ solutions for all^8,77^.

## Blue growth and the future of ‘blue foods’

In this final section, we conclude by addressing two sets of questions. First, why have these narratives emerged as a cohesive whole at this time, and what purpose do they serve? Second, where does the future of aquatic food production, including that with the greatest potential to contribute to food and nutrition security lie?

The narratives summarized in this paper intersect with and converge around the wider policy discourse of ‘blue growth’, which heralds the oceans as a new frontier for investment across multiple economic sectors^11,12,78,79^. An upsurge in interest in the ocean economy has encouraged the formation of new coalitions of actors and revitalized longstanding claims about the potential of marine aquaculture. This dynamic is illustrated by a recent report published by a large environmental NGO and an ‘impact investment firm’, that estimates that the aquaculture sector will require an additional USD 150–300 billion in capital investment by 2030. The report states that it seeks to catalyze greater investment into more sustainable marine aquaculture, to “create alternatives to wild caught fisheries and more resource intensive forms of land-based protein production while ensuring protection of marine ecosystems”^38^.

Many of the papers cited here are a product of similar new institutional alliances, based on academic research contributed to, or financially supported, by leading environmental NGOs, or produced with funding from philanthropies and foundations that promote marine conservation^1,5,6,8,10,19,25,28,29,66,76,77,80,81^. Research co-produced in this way includes papers advancing many of the most spectacular and newsworthy of claims. These articles provide much of the content for recent influential policy documents promoting the blue economy^3,4^.

This conjuncture suggests that some marine conservation groups, given a choice between marine aquaculture and capture fisheries, now consider aquaculture the lesser of two evils. These groups have formed alliances with academics, businesses, investors and governments attracted to marine aquaculture’s potential to advance a key goal: the zoning of marine space. This would enable the expansion of MPAs, while strengthening forms of governmentality necessary for other forms of extractive marine industry to flourish^82,83^.

In this way, the discourse of blue growth works to create conditions ripe for the appropriation of oceanic space for the benefit of private capital and conservation interests^17^. This trend mirrors well-documented processes of ‘green grabbing’ on land, under which, similar coalitions of actors advance crisis narratives that justify processes of financialization, state intervention, and enclosure and privatization of land and natural resources in the service of conservation and avowedly ‘green’ schemes such as carbon offsetting and biofuel production^16^. Blue economy initiatives led by state, commercial, and conservation interests, and associated forms of governmentality such as marine spatial planning are already contributing to the displacement of coastal fishers from customary fishing grounds^82,84,85^. Our interpretation is that the push to expand marine aquaculture is part of attempts by these actors to lay claim to and/or intensify the use of oceanic space and resources^79^.

Turning to the future of aquatic foods and their contributions to food and nutrition security, our analysis of the literature indicates that the new wave of marine aquaculture literature rests on a series of questionable assumptions. Most farmed aquatic foods originate from land-based freshwater production systems that are not fundamentally resource constrained^36,86^. The extremely rapid growth of terrestrial aquaculture over the past three decades shows little sign of abating in volume terms^36^. Much recent growth in production has occurred through intensification rather than horizontal expansion, enabling higher levels of farm productivity per unit land and water^45^. For example, Vietnam’s entire production of pangasius catfish, which stands at around one million tonnes per year, takes place on an area of 6000 hectares, equivalent to just 0.08% of the country’s total area of rice fields^87^.

The growth of terrestrial aquaculture has made farmed freshwater fish widely available and accessible to low- and middle-income consumers in countries that account for the majority of the world’s aquaculture production^45^, and makes important contributions to food and nutrition security by complementing fish supplies from inland and marine capture fisheries^88^.

As established above, the bio-economics of finfish production in offshore marine environments necessitates production of expensive carnivorous species. These cannot compete on price with relatively cheap herbivorous and omnivorous freshwater fish. Low trophic level marine finfish with similar price profiles to carp, catfish and tilapia do exist. Milkfish and mullets, farmed mainly in coastal ponds, contribute significantly to food and nutrition security in countries including the Egypt, Indonesia, and the Philippines^89–91^. Such modes of farming lack the techno-futuristic cachet of offshore cages, and do not require the establishment of new private property regimes. They are virtually absent from the accounts of marine aquaculture proponents.

Recent developments in the salmon industry suggest that closed intensive land-based recirculating aquaculture systems (RAS) are emerging as a viable alternative to cage-based systems. Unlike marine cages, RAS offer high levels of biosecurity and the ability to capture and utilize 100% of nutrient wastes. Advances in renewable energy production seem set to reduce RAS operating costs^92,93^, though farming will still likely be restricted to high value products.

Coastal fisheries already play an extremely important role in providing livelihoods^14,94^ and readily accessible nutritious aquatic foods^14,15,58^ in the Global South. By reframing offshore marine aquaculture as a viable source of food for the nutritionally vulnerable, blue growth policy discourse may appear to obviate the need for coastal fishers, and has the potential to justify their exclusion from MPAs and new zones of maritime economic activity, with results that are potentially highly inequitable.

We conclude that marine finfish aquaculture in offshore environments will confront economic, biophysical, and technological limitations that hinder its growth and prevent it from contributing significantly to global food and nutrition security. Nearshore marine finfish aquaculture is likely to continue expanding in some locations, but will mainly supply expensive products that contribute little to food and nutrition security or environmental sustainability, and perhaps undermine them. Capture fisheries are set to continue to be the source of most food from the oceans for the foreseeable future. The evidence suggests that freshwater aquaculture has far greater potential to continue to supply most of the world’s farmed aquatic food and contribute to human equity and food security than marine finfish farming. Policies and investments that seek to increase the availability and accessibility of affordable and sustainable farmed aquatic foods should look to the land^95^.
